# Smartphone-Based Platforms for Clinical Detections in Lung-Cancer-Related Exhaled Breath Biomarkers: A Review

**DOI:** 10.3390/bios12040223

**Published:** 2022-04-08

**Authors:** Qiwen Yu, Jing Chen, Wei Fu, Kanhar Ghulam Muhammad, Yi Li, Wenxin Liu, Linxin Xu, Hao Dong, Di Wang, Jun Liu, Yanli Lu, Xing Chen

**Affiliations:** 1 Biosensor National Special Laboratory, Key Laboratory for Biomedical Engineering of Education Ministry, Department of Biomedical Engineering, Zhejiang University, Hangzhou 310027, China; yuqw@zju.edu.cn (Q.Y.); wei.fu@zju.edu.cn (W.F.); kanhargm@zju.edu.cn (K.G.M.); liyijoy@zilai-tech.com (Y.L.); liuwx@zju.edu.cn (W.L.); xlinx@zju.edu.cn (L.X.); liujun@zju.edu.cn (J.L.); 2 School of Medical Technology and Information Engineering, Zhejiang Chinese Medical University, Hangzhou 310051, China; keichen@zju.edu.cn; 3 Research Center for Sensing Materials and Devices, Zhejiang Lab, Hangzhou 311100, China; cnhaodong@zhejianglab.com (H.D.); diwang@zhejianglab.com (D.W.)

**Keywords:** smartphone, volatile organic compounds, exhaled breath condensate, lung cancer

## Abstract

Lung cancer has been studied for decades because of its high morbidity and high mortality. Traditional methods involving bronchoscopy and needle biopsy are invasive and expensive, which makes patients suffer more risks and costs. Various noninvasive lung cancer markers, such as medical imaging indices, volatile organic compounds (VOCs), and exhaled breath condensates (EBCs), have been discovered for application in screening, diagnosis, and prognosis. However, the detection of markers still relies on bulky and professional instruments, which are limited to training personnel or laboratories. This seriously hinders population screening for early diagnosis of lung cancer. Advanced smartphones integrated with powerful applications can provide easy operation and real-time monitoring for healthcare, which demonstrates tremendous application scenarios in the biomedical analysis region from medical institutions or laboratories to personalized medicine. In this review, we propose an overview of lung-cancer-related noninvasive markers from exhaled breath, focusing on the novel development of smartphone-based platforms for the detection of these biomarkers. Lastly, we discuss the current limitations and potential solutions.

## 1. Introduction

Lung cancer (LC) is the most commonly diagnosed cancer, as well as the leading cause of cancer death in both sexes around the world [1]. Unfortunately, more than half of lung cancer patients are diagnosed at an advanced stage rather than being controlled at a more treatable status, due to the physiological symptoms of lung cancer being difficult to find in the early stages. The 5-year survival rate can be improved from 20% to 70% if lung cancer is discovered at stage I [2,3]. Effective methods for early and accurate diagnosis of lung cancer are urgently needed.

Lung cancer, as a chronic disease, develops over a long period of time. The diversity and complexity of tumor biological behavior lead to gradual changes in metabolism and genome to a certain extent during carcinogenesis. To date, thousands of markers related to lung cancer have been studied, identified, and applied to determine stages of cancer progression and track the carcinogenesis process of lung cancer. Biomarkers of metabolism involving VOCs, proteins, and genes can reflect cellular, biochemical, and molecular (proteomic, genetic, and epigenetic) alterations and help recognize and monitor normal or abnormal biological processes, which can be applied in the screening, diagnosis, evaluation of treatment, and monitoring of recurrence of lung cancer. Thus, metabolic and genetic biomarkers may offer a way to track, monitor, and evaluate the carcinogenesis process in a more precise way [4,5,6]. Physical characteristics such as shape, density, and position of nodules in lung tissue imaged by chest X-ray, computer tomography (CT), magnetic resonance imaging (MRI), and positron emission tomography (PET) have been regarded as effective means of cancer in diagnosis [7]. However, these conventional instruments are relatively expensive, bulky, and limited to trained personnel, which greatly constrains their application in the field of population screening for early diagnosis. The development of novel technologies such as breath biopsy will allow us to detect cancer in the early stages and better identify the process of tumor cell growth and cellular metabolism. Detection of biomarkers in exhaled breath is one of the most promising methods due to its high-throughput detection, low cost, and noninvasiveness. According to different collection procedures, exhaled breath can be divided into a gas phase that is made of VOCs and a liquid phase, which is the condensate part of the breath containing both volatile and nonvolatile compounds [3,8,9]. Some techniques, such as gas/liquid chromatography (GC/LC) and mass spectrometry (MS), have been shown as a gold standard [10] in detecting VOCs and EBCs carried on human breath. However, despite significant improvements in sensitivity and accuracy, chromatography and spectrometry still have the aforementioned demerits, which hinders the wide range of application.

**Figure 1:**
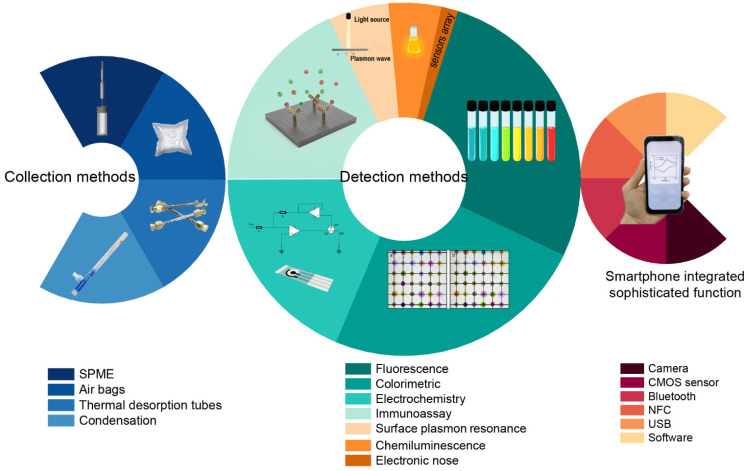
Schematic representation of smartphone-based biomarker detection platforms from sample collection to detection. Pie charts representing proportions of smartphone-based detection methods with different technologies.

Recently, novel technologies based on smartphones have been developed to satisfy the demand for low cost, miniaturization, and easy operation. The smartphone is a highly integrated intelligent device that plays an irreplaceable role in our daily life due to its robust function, including a high-resolution camera, complementary metal-oxide semiconductor (CMOS) sensors, Bluetooth, near-field communication (NFC), universal serial bus (USB), and so on. Thus, as an ideal handheld device, a smartphone can provide a more convenient and fast response for healthcare detection. For example, smartphone-based electronic nose sensors, including quartz microbalances, surface acoustic wave sensors, and gold nanoparticles, have displayed great improvement in the analysis procedures for early diagnosis of diseases [11]. In addition, combined with smartphones, immunoassay analysis for the detection of genes and epigenetic markers such as DNA methylation and microRNAs in EBCs is also a promising method for lung cancer diagnosis [12]. These diagnostic procedures show high, broad relevance. This review discusses lung-cancer-related biomarkers in exhaled breath and smartphone-based methods of detection, providing an overview of the recently developed technology for breath biopsy that can likely be utilized in future clinical practice. Figure 1 summarizes the common collection methods for VOCs and EBCs, both in the laboratory and commercial platforms, focusing on smartphone-based detection technologies and the sophisticated function of smartphones for use in detection procedures.

## 2. Smartphone-Based Detection Methods

### 2.1. Optical Sensors

#### 2.1.1. Colorimetric Sensors

**Figure 2:**
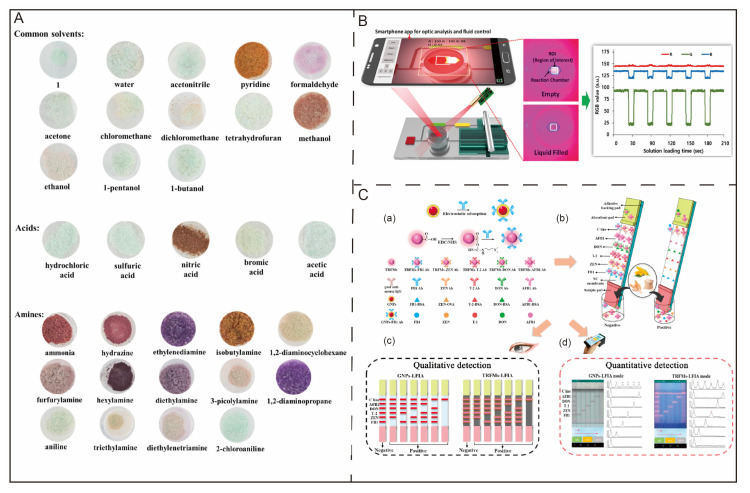
(**A**) Schematic of vapochromic transformation for the sensing of VOCs by Complex 1. (**B**) Illustration of the optofluidic automated 96-well LOC device. Incident light on the reaction chamber of the microfluidic device was captured by a camera, and the RGB value of ROI was analyzed with a smartphone. (**C**) Schematic diagram of smartphone-based GNPs and TRFMs-LFIAs for mycotoxin detection. (**a**) Preparation of immune probes; (**b**) detection principle of test strips; (**c**) qualitative test results with the naked eye; (**d**) quantitative test results with smartphones.

Rapid iterative updates to complementary metal-oxide semiconductor (CMOS) sensors have further accelerated the optical analysis of smartphones without additional peripherals, especially in colorimetry. The design of colorimetric sensor arrays is based on dye–analyte interactions more powerful than simple physical adsorption [13]. These arrays are composed of a diverse set of chemically responsive colorants, including metalloporphyrins and chemically responsive dyes. Mazzone et al. [14] showed that patients with lung cancer had unique chemical signatures on their breath, which could be detected by a colorimetric sensor array with moderate accuracy. Recently, Sun and coworkers [15] reported a novel, fast, and portable colorimetric chemosensor integrated with a smartphone for in situ analysis of gas-phase VOCs and hazardous gases (HGs). This chemosensor, based on [Fe(H_2_btm)_2_(H_2_O)_2_]Cl_2_ (1) (H_2_btm = di(1*H*-tetrazol-5-yl)methane) with a detectable color change when discriminating VOCs and HGs, could be observed by the naked eye under ambient conditions. Figure 2A shows various vapochromic transformations for the sensing of VOCs by Complex 1. Finally, a mobile application was introduced to correlate VOC concentrations and Euclidean distance. The Euclidean distance corresponded to the total length of six-dimensional color vectors, red, green, and blue (RGB) and hue, saturation, and brightness (HSB) values, and was collected via a smartphone.

#### 2.1.2. Immunoassay Sensors

Labeled immunoassay technology is a general term that describes a type of detection technology based on immunoassay with ultrahigh sensitivity and high specificity in the determination of trace bioactive substances. Many methods have been reported in the biomedical literature, and most have been developed based on radioimmunoassay (RIA) [16], enzyme immunoassay (EIA) [17], chemiluminescence immunoassay (CLIA) [18], and time-resolved fluorescence immunoassay (TRFIA) [19]. The history of immunoassays goes back to the 1950s, when immunoassay was first used to detect biomacromolecules. Yalow and Berson combined the isotopic tracer technique with an immunoassay to detect the concentration of insulin [20] in the 1960s, which became the foundation for radioimmunoassay. Although radioactive contamination has troubled people, RIA has not been eliminated due to its advantages of simple operation and low cost, which can reduce the financial burden on patients.

EIA is a nonradioactive marked immunoassay technology that was developed based on RIA. Due to the characteristics of easy preparation, long-term validity, and lack of pollution to the environment, EIA has been widely applied. EIA can be classified into two different types of assays, including heterogeneous and homogeneous enzyme immunoassays [21]. The distinction between these two methods is that homogeneous enzyme immunoassays achieve the quantitative determination of analytes by enzyme activity [22]. Heterogeneous enzyme immunoassays include the enzyme-linked immunosorbent assay (ELISA), which is widely used in clinical settings. ELISA can be used to determine antigens and antibodies. Different detection methods can be designed according to the source of reagents, the characteristics of specimens, and the conditions for detection. In addition, lab-on-a-chip (LOC) devices or microfluidic systems have seen huge advances in biomolecule diagnostics and biosensing technologies. In accordance with this concept, Uddin et al. [23] utilized active 96-well hybrid lab-on-a-chip (LOC) microfluidic devices (Figure 2B) to execute ELISA to determine the NT-proBNP human cardiac biomarker with a low detection limit of 7.81 pg mL^−1^. A smartphone was introduced for optical analysis and as a controller.

Time-resolved fluorescence immunoassay (TRFIA) technology, a fluorescence-based assay, is another nonradioactive immunoanalysis method developed in the 1980s. Time-resolved fluorescence analysis is an ultrasensitive detection technique characterized by a rare earth ion labeled antigen or antibody, nucleic acid probe, and cell, among other elements. It overcomes the disadvantages of enzyme markers such as instability; chemiluminescence, which can only emit light once and is easily disturbed by the environment; and electrochemical luminescence, which is not directly labeled. Its main characteristic is that lanthanides, such as Eu^3+^, Dy^3+^, Tb^3+^, or Sm^3+^, serve as tracers. Lanthanides have a large Stokes shift, allowing operators to easily distinguish excitation and emission and exclude excitation light interference [24]. Based on this property, Liu et al. [25] established a fast-response and high-sensitivity smartphone-based quantitative dual-detection-mode device for the quantification of multiplex mycotoxins. In this study, time-resolved fluorescence microspheres (TRFMs, europium chelate), lateral flow immunoassays (LFIAs), and gold nanoparticles (GNPs) were used as the corresponding signal labels to achieve fluorescence and visible-light detection modes. In this developed detection device (Figure 2C), fluorescence and visual light were captured by a cell phone camera and analyzed with early established standard curves of the five mycotoxins.

**Figure 3:**
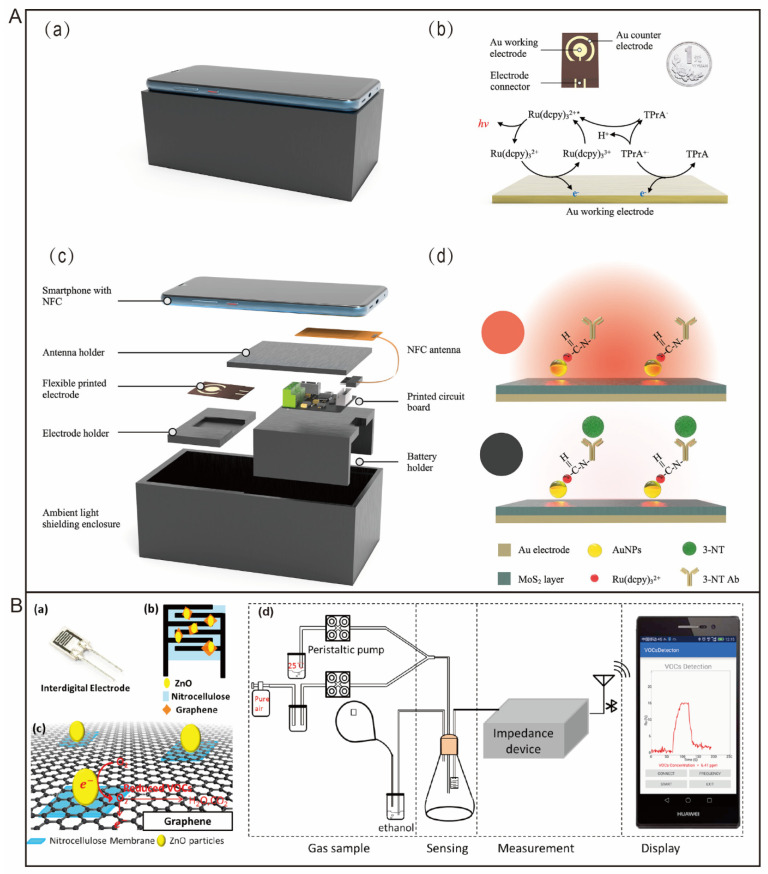
(**A**) Schematic diagram of smartphone-based ECL analysis device. (**a**) Three-dimensional (3D)-printing shell integrated with smartphone excluding ambient light. (**b**) Principle of Ru(dcpy)32+/TPrA-based ECL. (**c**) Details of the device in exploded view. (**d**) Diagram of ECL analysis for 3-nitrotyrosine detection. (**B**) Schematic diagram of smartphone-based sensor for VOC detection. (**a**) Photo of the interdigital electrodes. (**b**) Electrodes modified by graphene, ZnO, and nitrocellulose membrane. (**c**) Mechanism for detection of VOCs on the modified electrodes. (**d**) Schematic diagram of detecting procedures, including gas sample, sensing, measurement, and display on a smartphone.

Building on the basic theory of RIA and EIA, CLIA technology was built from a chemiluminescence signal tracer in 1978 and perfected by British researchers from an Amersham company in the early 1990s. The widely utilized test kit was then developed and produced. It has similar sensitivity and specificity to RIA but no radioactive nuclide contamination. Chemiluminescence, with the advantages of high sensitivity, is still limited to bulky instruments such as photomultiplier tubes (PMTs) and fiber-optic spectrophotometers. In 2019, Li and colleagues [26] used graphene quantum dot (GQD) nanocomposites to amplify electrogenerated chemiluminescence (ECL) signals to detect *Escherichia coli* (*E. coli*). The ECL signals were captured by a smartphone camera and processed by a smartphone-based image analysis application (APP). The smartphone-based ECL system provided potentiostatic control via a universal serial bus (USB) port without an external power supply. Similarly, a handheld electrochemiluminescence immunoassay (ECLIA) analysis device was developed for 3-nitrotyrosine detection [27]. As shown in Figure 3A, this device reduced the size of the ECL analysis system into a 3.5 cm × 4.0 cm printed circuit board (PCB) and realized wireless, short-range communication by near-field communication (NFC) from a smartphone. Antibody/Ru(dcpy)_3_^2+^@AuNPs/MoS_2_-modified Au electrodes (Ab/Ru@AuNPs/MoS_2_) were utilized for 3-nitrotyrosine detection.

### 2.2. Electrochemical Sensors

Electrochemistry has been widely explored in biomolecule detection and quantification due to its advantages of low cost, easy operation, and reliability. When integrated with smartphone platforms, electrochemical sensors have achieved great success in point-of-care testing. There are mainly three classes of smartphone-based electrochemical sensors: amperometric biosensors, potentiometric biosensors, and impedimetric biosensors [28]. Most smartphone-based electrochemical sensors rely on an extra circuit board to provide potentiostatic and galvanostatic stability. Smartphones often act as readers and processors. Liu et al. [29], using ZnO and graphene as sensitive materials, developed a smartphone-based sensing system for VOC recognition from human breath (Figure 3B). The electrode was designed into an interdigitated structure for impedance transduction. As a result, VOCs reacted with the surface of the modified electrode, resulting in changes in the electrical properties, which were quantized by a handheld wireless device designed for impedance measurement.

### 2.3. Electronic Noses

There are many types of sensors and sensor array techniques, collectively known as electronic nose systems. An electronic nose is a type of sensor technology used for the quick detection of breath. Although new technologies have recently entered this field, the classical electronic nose, which consists of an array of sensors, is still the most common approach [30]. Here, we focus on common sensors and sensor arrays, such as metal-oxide (MOX) and surface acoustic wave (SAW) sensors, which are combined with smartphones in biomarker detection.

A gas sensor array (GSA) is composed of several gas sensors with different response characteristics to a specific analyte. Researchers used an electronic nose comprising several MOX sensors to distinguish lung cancer patients from healthy subjects. Each sensor adopted an individual molecular recognition element and translated different chemical signals into electrical signals for real-time detection. Additionally, conducting polymer (CP) microsensors [31] are commonly used due to their high stability, sensitivity, and selectivity. The physical and electrical properties of certain conducting polymers can be altered by some specific analytes. Thus, MOX sensor arrays and CP sensor arrays have been widely used in commercial e-nose systems.

Additionally, surface acoustic wave (SAW) sensors are mainly composed of a piezoelectric substrate and input (and output) interdigital transducers (IDTs) that are deposited on the substrate [32]. A sensing material is fixed between two interdigital electrodes. When gas components adhere to the sensitive material, the load quality of the sensitive areas will increase, leading to a change in the acoustic transmission path, causing a change in the acoustic characteristics (e.g., acoustic transmission speed, amplitude, and frequency) [33]. By measuring the change in the frequency or phase, the concentration or partial pressure of gas components can be directly obtained or indirectly calculated. Guo et al. [34] proposed a novel uncoated surface acoustic wave resonator (SAWR) sensor system integrated with a gas chromatographic capillary column to determine VOC constituent parts. A smartphone provided a real-time display and analysis of the frequency data via Bluetooth communication. Compared with other sensors, smartphone-based SAW sensors have the advantages of high sensitivity, short response time, low power consumption, small size, and high stability.

## 3. Biomarkers in VOCs and EBCs

### 3.1. Breath Sampling and Preconcentration

Exhaled breath is composed of air from the mouth, nasal cavity, alveoli, and part dead space. Biomarkers associated with different diseases are produced in different locations. Exhaled breath biomarkers that have been fully exchanged in the lung–blood circulatory system will be comparatively accurate in their reflection of lung diseases and metabolic diseases. Although there are high-sensitivity gas sensors [35] that have achieved rapid online detection of components in exhaled breath, the most accurate detection of exhaled breath components still requires enrichment before detection and analysis. Therefore, the collection and storage of respiratory samples have an impact on subsequent procedures. However, there is still no standardized sampling protocol for collecting patient exhalation. In addition, the concentration of exhaled breath markers is between the volume fraction ppb and ppt. This concentration level is commonly lower than the limit of detection of equipment during direct detection of exhaled breath. Furthermore, the complete elimination of gas from unexchanged dead space and the oral and nasal cavities is still a challenge.

Thus, researchers have mostly chosen to collect late expiratory samples to eliminate dead space and have used polymer bags as collecting sample containers [36] due to the advantages of low cost and easy operation. Normally, subjects are asked to take a deep breath, and the first collected breath is discarded. Additionally, some researchers stipulated strict sampling procedures. Chang et al. [37] asked subjects not to eat, smoke, brush their teeth, or gargle for a minimum of 2 h before sampling. Ambient air is another interference that can greatly alter analysis results. Di Gilio et al. [38] required that volunteers waited in the room for at least 10 min before breath collection so that equilibrium could be created between the lungs and ambient air.

Owing to the low concentration of VOCs in exhaled breath [39], preconcentration is commonly used to improve the accuracy and sensitivity of detection. Preconcentration methods include solid-phase microextraction (SPME), thermal desorption enrichment technology, needle trap microextraction (NTME), and so on. In essence, most methods use the force (such as van der Waals forces) between adsorption materials and gas molecules at the microscopic scale. The flexible use of these enrichment methods can not only eliminate more than 90% of the water vapor in exhaled breath but also greatly enrich the target markers by 10- to 100-fold. SPME is easy to use and widely used in many environmental, clinical, and biological analyses. Normally, SPME fibers are coated with various polymers such as polydimethylsiloxane, divinylbenzene, polyacrylate, and polyethylene glycol [40] to absorb analytes within the ppb range. Thermal desorption enrichment technologies mainly rely on adsorption materials. The adsorbents used in breath testing include carbon nanotubes, Tenax^®^ TA, carbon black, and graphene. Due to its low breakthrough volume for water and high efficiency in the adsorption of volatile gas molecules, Tenax^®^ TA is widely used in expiratory enrichment equipment. Tenax^®^ TA is a 2,6-diphenyl furan resin polymer with a structure of porous microspheres that have a large specific surface area to adsorb target substances effectively. In addition, the filling of layered adsorbents can simultaneously concentrate multiple types of gas markers. The filled adsorbent tube needs to be heated at high temperatures in a flow of inert gas before use. It is worth noting that different materials have different desorption temperatures, and specific chemicals can be preserved by this technique, while others cannot [41].

EBC collection realized by condensing exhaled breath in a container in a low-temperature environment is easier than VOC sampling. Normal exhalation is saturated with water vapor at a temperature of 37 °C. When the external temperature is 0 °C and −10 °C, 89.1% and 93.7% of water vapor are condensed, respectively. Airway lining fluid, proteins of small relative molecular mass, and volatile compounds are exhaled together in quiet breathing. When the temperature is reduced, nonvolatile substances can condense into EBCs.

### 3.2. Biomarkers in VOCs

More than 1000 volatile organic compounds (VOCs) have been identified in human breath [42]. Lung-cancer-correlated VOCs have been sought in case–control studies and verified by cohort studies. To date, seven categories of VOCs have been found to serve as biomarkers of lung cancer in exhaled breath. The categories are alkanes, alkanols, aldehydes, ketones, lipids, nitriles, and aromatics. Researchers thought that alkanes such as ethane and pentane were generated by the lipid peroxidation of polyunsaturated fatty acids in cell membranes [43,44,45]. Breath methyl alkanes may also be products of the same process [46]. The first report on exhaled VOCs in lung cancer dates back to 1985, when Gordon et al. identified that several VOCs in exhaled breath were associated with lung cancer [47]. In 1999, Phillips et al. used a combination of 22 VOCs in breath samples to distinguish between patients with and without lung cancer [48]. Poli et al. demonstrated that a combination of 13 VOCs could be used to correctly classify lung cancer patients, chronic obstructive pulmonary disease (COPD) patients, asymptomatic smokers, and healthy subjects into defined groups [49]. Additionally, it is worth noting that some pulmonary diseases, especially COPD or emphysema, could be a major risk factor for lung cancer. This is because COPD and lung cancer share common risk factors such as smoking [50]. Wehinger et al. reported that the concentration of mass-to-charge ratios 31 and 43 were increased in lung cancer patients in a study using proton-transfer-reaction mass spectrometry (PTR-MS) [51]. Table 1 summarizes the discovery of VOC markers related to lung cancer.

**Figure 4:**
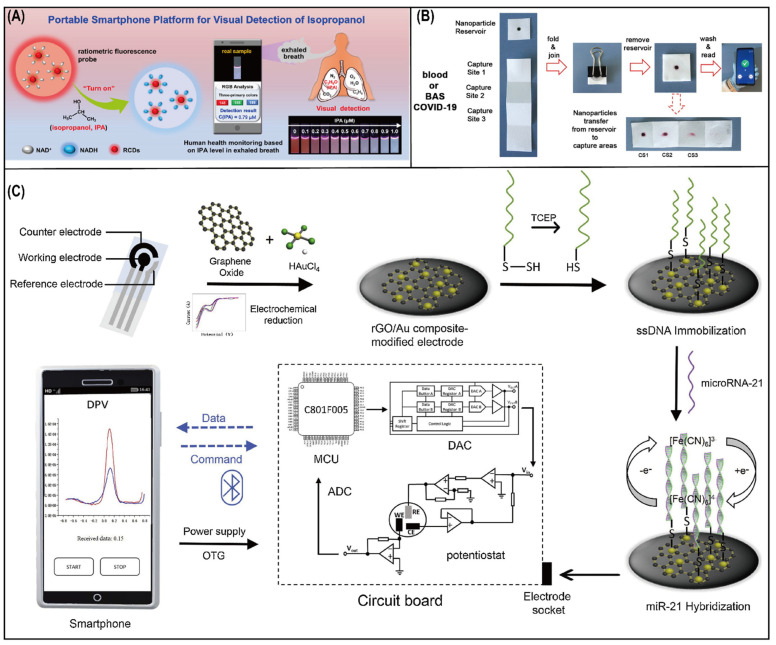
Schematic diagram for detection of IPA, IL-6, and microRNA using a smartphone. (**A**) Schematic diagram of the ratiometric fluorescence sensing system for IPA detection and RGB analysis of the fluorescent photo captured by the smartphone. (**B**) Diagrammatic representation of the paper immunosensor and analytical steps for detecting IL-6 in blood or respiratory samples (bronchial aspirate (BAS)) from COVID-19 patients. (**C**) Illustration of smartphone-based electrochemical biosensing system, including reduced graphene oxide/gold composite-modified electrode, circuit board, and smartphone with Android application.

Based on the aforementioned techniques, smartphones provide promising potential in lung-cancer-correlated VOCs such as alkanols and ketones. For instance, Salimi and colleagues [62] introduced a smartphone-based chemiresistive device using a zinc oxide nanosheet that could detect diethyl ketone (LOD = 0.9 ppb), acetone (LOD = 4 ppb), isopropanol (IPA, LOD = 11 ppb), and other alcohols that are related to lung cancer. Most recently, a fluorescence-based smartphone platform was reported for real-time/on-site, sensitive, and quantitative visual detection of IPA in exhaled breath [63]. In this approach, red carbon dots (RCDs) were modified by a coenzyme (nicotinamide adenine dinucleotide, NAD^+^) for fluorescence, as shown in Figure 4A. This NAD-dependent enzymatic reaction provided the electron transfer from IPA to NAD^+^ and displayed continuous color changes from red to light blue, which could be detected and captured by a smartphone. The detection limit and recovery rate of the fluorescence-based smartphone platform were 8.34 nM and 90.65–110.09% (RSD ≤ 4.83), respectively.

### 3.3. Biomarkers in EBCs

EBCs also provide a convenient and noninvasive method for the diagnosis of lung cancer. EBCs, which are formed by cooling exhaled breath, contain low-volatility or nonvolatile markers that reflect the status of the airway lining fluid (ALF) environment [8]. Many biomarkers have been reported in EBCs. Carpagnano et al. indicated that the concentrations of inflammatory markers such as cytokines including interleukin-6 (IL-6) and vascular stimulating factor (endothelin-1) were significantly different in the EBCs of patients with lung cancer than controls, and the same difference between the various stages of NSCLC was also found. In addition to the cytokines mentioned above, oxidative-stress- and lipid-peroxidation-related factors, including 8-isoprostane F2α, hydrogen peroxide (H_2_O_2_), etc., were also found in EBCs [64].

More interesting data have been acquired by researching genetic markers in EBCs. Microsatellite DNA alterations involving carcinogenesis and tumor progression are valuable as clonal markers for the detection of cancers. Carpagnano et al. demonstrated the possibility of studying microsatellite alterations (MAs) of 3p in the DNA of EBCs in patients with NSCLC. They concluded that MAs were significantly more frequent in NSCLC patients than in control subjects. In addition, epigenetics has become increasingly important in the early diagnosis and treatment of lung cancer. DNA methylation, histone modification, and long noncoding RNA (lncRNA) can all be used as biomarkers for lung cancer detection [65,66]. Han et al. showed that DNA methylation in EBCs is detectable, and that the DNA appears to be of lower airway or lung origin and has some association with lung cancer and smoking [67]. Epigenetic markers can be found in blood, serum, EBCs, phlegm, and alveolar analytes [68], reducing the invasiveness of sampling. Considering the integrality of this marker in the field of cancer studies, DNA analysis in EBCs seems to be a strong screening tool.

The detection of markers in EBCs is always performed using marked immunoassay technology for protein markers and polymerase chain reaction (PCR) for genetic markers. On the basis of a marked immunoassay, Zhang et al. developed a love-wave surface acoustic wave (LWSAW) immunosensor to measure proteins in EBCs. They reported a miniaturized platform that provides online detection of CEA in EBCs. The detection method adopted here was a sandwich immunoassay using AuNP–antibody conjugates with a gold-staining signal enhancement strategy. This platform displayed a good performance in the measurement of CEA concentrations, with a limit of detection of 1.25 ng/mL and a coefficient of determination (r^2^) of 0.998 [69]. In addition, liquid chromatography–mass spectrography (LC–MS) is also very popular with many researchers [70,71]. However, some limitations impede the application of LC–MS in the fast analysis of exhaled breath. The complexity of operation and maintenance of LC–MS, especially in the preprocessing of samples, introduce a stringent requirement for operators. Additionally, the expensive cost and large size of this instrument might deter small institutions. Thus, smartphone-based LOC systems involving portability, low cost, easy operation, and relatively high sensitivity and specificity have attracted the attention of researchers.

#### 3.3.1. IL-6

Interleukin-6 (IL-6), as a cytokine, plays a crucial role in immune response, inflammation, and cell proliferation. Clinical investigations [72] found increased serum levels of IL-6 were associated with lung cancer and lung cancer risk. Conventional immunoassay protocols were used for determination. Recently, a paper-based immunosensor integrated with a smartphone was fabricated to rapidly detect increased IL-6 levels in blood and respiratory samples from COVID-19 patients [73]. The devices of this paper (Figure 4B) were composed of two parts: a paper square and a paper strip. The paper square was modified with polystyrene sulfonate (PSS) reservoirs containing antibody-decorated nanoparticles, and the paper strip was used to capture target analytes with three capture sites. For IL-6 detection, samples were successively added to the sites, and then the strip was folded and soaked with a blocking solution. The reservoir was subsequently pressed on top of the folded strip using a clamp. With the help of the clamp, antibody-decorated nanoparticles infiltrated the folded strip to the three capture sites and reacted with IL-6. Then, a colored spot directly corresponded with the concentration of IL-6 in the sample. Afterwards, amplified colorimetric signals were recorded by a smartphone, and a developed app was installed on the smartphone for the quantification of IL-6.

#### 3.3.2. Microsatellite DNA

Microsatellite DNA alteration markers have shown good specificity and sensitivity in the early detection of cancers [74]. Studies have shown that the development of NSCLC is closely related to genome-wide DNA methylation [75]. As one of the earliest and most deeply studied epigenetic mechanisms, DNA methylation detection methods are varied [65], including methylation-specific PCR (MSP) [76]. For instance, Kalligosfyri et al. [77] reported a smartphone-based chemiluminometric quantitative competitive PCR for the detection of DNA. Smartphones, as luminescence detectors/imagers, provide rapid acquisition, high detectability and reproducibility, low cost, and high portability potential for DNA detection. The LOD was calculated to be 1.6 pM.

#### 3.3.3. MicroRNAs

MicroRNAs are another type of marker used in the diagnosis of cancer. MicroRNAs are tiny noncoding RNA molecules that play an important role in the epigenetic control of cellular processes and can specifically bind to target messenger RNAs (mRNAs). MicroRNAs can prevent the translation of proteins from mRNAs. The ability of microRNAs to influence the expression of hundreds of different mRNAs can be used to help identify novel biological pathways. Changes in microRNA expression have been associated with a wide variety of disease conditions; thus, microRNA-based biomarkers can be developed to identify and monitor such states [78,79]. Studies have demonstrated that microRNAs can be detected in serum or plasma, as well as in EBCs [79]. Researchers [80] measured microRNA-21 and microRNA-486 expression in plasma and EBC samples from patients with NSCLC and controls. They found that the AUC of both microRNA-21 and microRNA-486 showed a significantly different from 0.5, although the expression of microRNA-21 was significantly higher in the plasma and EBC samples of the NSCLC patients, while microRNA-486 was significantly lower. This result indicates not only that microRNAs can be clinically used in first-line screening tests in high-risk subjects but also that microRNAs can be detected in exhaled breath for the noninvasive diagnosis of lung cancer. Various smartphone-based methods, including colorimetric detection [81], electrochemical sensors [82], and luminescence detection [83,84] have been developed for microRNA detection in recent years. Among them, electrochemical sensors, with the advantage of low cost, high sensitivity, and accuracy, are a strong alternative for miRNA detection. For instance, Low and coworkers [82] developed a portable and highly sensitive smartphone-based system for rapid detection of circulating microRNA-21 (miR-21) biomarkers in saliva. As shown in Figure 4C, a disposable SPCE was modified with reduced graphene oxide/gold (rGO/Au) composite for enhancing electrochemical performance. A printed circuit board (PCB) integrated with a Bluetooth-enabled smartphone was exploited to signal handling and process control. The TCEP-treated ssDNA probe on the modified working electrode was used to hybridize with the miR-21 target. The DPV peak current showed a negative correlation when adding different miR-21 target concentrations. The linear detection range of miR-21 was from 1 × 10^−4^ M to 1 × 10^−12^ M, with a recovery rate ranging from 96.2% to 107.2%.

To conclude, the above-mentioned technologies have provided the basic principles of smartphone-based detection methods in recent years. Additionally, Cheng et al. used field-effect transistor (FET)-based biosensors for detecting lung cancer biomarkers [85,86]. Moreover, studies have adopted aptasensors based on upconversion fluorescence resonance energy transfer [87] and aptamer-modified film bulk acoustic resonators to detect carcinoembryonic antigen (CEA) [88]. New detection methods are continuously emerging. Detection machines have been developed from bulky instruments such GC–MS to small-volume instruments such as smartphone-based GSAs, which are portable and handheld. The decrease in volume has meant that breath tests can spread to family, community, and clinical settings.

## 4. Conclusions and Perspectives

Many researchers are contributing to the diagnosis of lung cancer. Precise targeting of lung cancer will not only lead to timely diagnosis and prompt treatment for patients but also bring them the benefits of reduced mortality and medical costs. In this review, we presented several lung-cancer-related biomarkers from exhaled breath and smartphone-based technological tools for the detection of these biomarkers, providing a potential method for fast, easy, and low-cost early screening or health monitoring. Smartphone-based platforms combine different sensors with high sensitivity, specificity, and accuracy; small size; low cost; and easy operation, which addresses the shortcomings of commercial bulky instruments such as GC–MS and LC–MS. Smartphone-based diagnosis platforms have shown tremendous potential in clinical applications, but most of them are still incubated in laboratories due to a number of additional challenges. Firstly, a portable peripheral device such as a sensitive front end integrated with a smartphone is necessary to complete the detection procedures. Smartphones are mostly designed as data transmission carriers for immunoassay and electrochemistry analysis. Despite the ubiquity of smartphones in modern life, there is no doubt that the rising detection cost, such as immune antibody refrigeration, and transportation, limit further use by the general public. Thus, researchers have attempted to reduce the detection cost and miniaturize front-end sensors as small as possible to broaden immuno- and electrochemical-based applications. For example, Xu et al. [89] recently developed a battery-free, wireless, and epidermal electrochemical system to detect metabolites in sweat by adopting NFC and a stretchable electrochemical electrode array. Smartphones with an NFC module show great functions in wireless power and data transmission. This miniaturized system is a broad-spectrum platform that can be used to analyze various biofluids. Secondly, a major concern for smartphone-based devices is the privacy and security of sensitive information of patients. In our investigation, most reported studies did not mention data security and measures to prevent data leakage. Smartphone-based technologies will be deeply integrated with clinics and the general public in the future, and data obtained from smartphone-based platforms may be related to more important and sensitive healthcare information. Robust and safe operating applications are needed to be built by developers and supervised by relevant institutions. Finally, limited by computational and statistical processing power, further data processing, such as principal component analysis (PCA), clustering analysis (CA), and artificial neural network analysis (ANNs), are still unapplied in smartphones. This may reduce the accuracy and efficiency of identifying biomarkers for diagnosis, especially in disease classification such as lung cancer in its early stages.

So far, thousands of markers related to lung cancer have been continuously studied, discovered, and applied. However, the diversity and complexity of tumor biological behavior make it difficult to find a single marker with high sensitivity and specificity simultaneously. Therefore, the combination of different markers could be an optional choice to improve clinical effectiveness in the diagnosis of lung cancer. For example, the combination of medical imaging and metabolic, genetic, and protein biomarkers may provide a more precise and prospective diagnosis of lung cancer at an early stage. Smartphones can be an excellent vehicle for daily screening by people themselves. On the other hand, behavioral imprinting can be applied for different purposes of detection. We have conventionally regarded people with a long smoking history and whose age is more than 50 as a high-risk population for lung cancer [90]. Therefore, smartphone applications that can record behaviors can make people at high risk pay more attention to their health status and may reduce the cases of lung cancer at late stages due to the high frequency of diagnoses.

New markers will be continually discovered by scientists, and different combinations of advanced technologies will be studied to enable better diagnoses of lung cancer in the future. In addition, detection technology for markers will be developed into machines that are more rapid, accurate, robust, and comfortable for the diagnosis of lung cancer.

## Figures and Tables

**Table 1:** Reported exhaled VOC markers for the early screening of lung cancer.

Years	Author	Collection Method	Sample	VOCs
1985	Gordon [47]	Tenax GC sorbent cartridges	Expired breath	Acetone, 2-butanone, n-propanol
1999	Phillips [48]	Sorbent trap	Alveolar breath	Styrene, 2,2,4,6,6-pentamethylheptane, 2-methylheptane, decane, n-propylbenzene undecane, methyl cyclopentane, 1-methyl-2-pentylcyclopropane, trichlorofluoromethane, benzene, 1,2,4-trimethylbenzene, isoprene, 3-methyloctane, 1-hexene, 3-methylnonane, 1-heptene, 1,4-dimethylbenzene, 2,4-dimethylheptane, hexanal, cyclohexane, 1-methylethenylbenzene, heptanal
2005	Poli [49]	Teflon^®^ bulb; SPME	Mixed expiratory samples	Isoprene; methylpentane; pentane; ethylbenzene; xylenes; trimethylbenzene; toluene; benzene; heptane; decane, styrene; octane; pentamethylheptane
2007	Wehinger [51]	Tedlar^®^ bags	Alveolar breath	Formaldehyde, isopropanol
2009	Bajtarevic [52]	Tedlar^®^ bags	Mixed expiratory and indoor air	Isoprene, acetone, methanol; 2-butanone, benzaldehyde, 2,3-butanedione, 1-propanol, 2-butanone, 3-hydroxy-, 3-butyn-2-ol, butane, 2-methyl-, 2-butene, 2-methyl-, acetophenone, 1-cyclopentene, methyl propyl sulfide, urea, tetramethyl-, n-pentanal, 1,3-cyclopentadiene, 1-methyl-, 2-butanol, 2,3-dimethyl-, isoquinoline, 1,2,3,4-tetrahydro-, undecane, 3,7-dimethyl-, benzene, cyclobutyl-, butyl acetate, ethylenimine, n-undecane,
2010	Fuchs [53]	Mylar sampling bag	Alveolar breath	p-Cymene, toluene, dodecane, 3,3-dimethylpentane, 2,3,4-trimethylhexane, (1-phenyl-1-butenyl)benzene 1,3-dimethylbenzene, 1-iodononane, [(1,1-dimethylethyl) thiol]acetic acid, 4-(4-propylcyclohexyl)-4′-cyano [1,1′-biphenyl]4-yl ester benzoic acid, 2-amino-5-isopropyl-8-methyl-1-azulenecarbonitrile, 5-(2-methylpropyl)nonane, 2,3,4-trimethyldecane, 6-ethyl-3-octanyl 2-(trifluoromethyl)benzoate, p-xylene, and 2,2-dimethyldecane
2010	Song [54]	Tedlar^®^ gas bags; SPME	Mixed expiratory samples	1-Butanol and 3-hydroxy-2-butanone
2011	Ulanowska [55]	Tedlar^®^ bags; SPME	Alveolar breath	Ethanol, acetone, butane, dimethyl sulfide, isoprene, propanal, 1-propanol, 2-pentanone, furan, o-xylene, ethylbenzene, pentanal, hexanal, nonane
2012	Buszewski [56]	Tedlar^®^ bags; SPME	Alveolar breath	Butanal, ethyl acetate, 2-pentanone, ethylbenzene, 1-propanol, 2-propanol
2015	Kumar [57]	Nalophan bag	Mixed alveolar breath	Pentanoic acid; hexanoic acid; phenol; methyl phenol; ethyl phenol; butanal; pentanal; hexanal; heptanal; octanal; nonanal; decanal
2016	Schallschmidt [58]	Gas bulbs; SPME	Tidal breath	Propanal, butanal, decanal, butanal, 2-butanone, ethylbenzene
2017	Sakumura [59]	Analytic Barrier Bag	Alveolar breath	Hydrogen cyanide, methanol, acetonitrile, isoprene, 1-propanol
2019	Phillips [60]	Carbotrap C and Carbopack C	Alveolar breath	1,4-Butanediol, 2-pentanamine, 4-methyl-, 2-propanamine, 3-butenamide, 4-penten-2-ol, acetamide, 2-cyanoalanine, n-methylglycine, octodrine
2019	Li [61]	Tedlar^®^ bags; SPME	End-tidal breath	Isopropanol, n-butanol, n-heptanol, n-hexanal, n-heptanal, n-decanal

## Data Availability

Not applicable.
